# Identifying Gravity-Related Artifacts on Ballistocardiography Signals by Comparing Weightlessness and Normal Gravity Recordings (ARTIFACTS): Protocol for an Observational Study

**DOI:** 10.2196/63306

**Published:** 2024-09-26

**Authors:** Urs-Vito Albrecht, Annabelle Mielitz, Kazi Mohammad Abidur Rahman, Ulf Kulau

**Affiliations:** 1 Department of Digital Medicine Bielefeld University Bielefeld Germany; 2 Smart Sensors Group Hamburg Technical University Hamburg Germany

**Keywords:** ballistocardiography, seismocardiography, acceleration, artifact, weightlessness, gravity, observational study, heartbeat, blood flow, intrinsic sensor, hypotheses, assessment, heart-induced, sensor, gyroscopes, cardiovascular, diagnostic

## Abstract

**Background:**

Modern ballistocardiography (BCG) and seismocardiography (SCG) use acceleration sensors to measure oscillating recoil movements of the body caused by the heartbeat and blood flow, which are transmitted to the body surface. Acceleration artifacts occur through intrinsic sensor roll, pitch, and yaw movements, assessed by the angular velocities of the respective sensor, during measurements that bias the signal interpretation.

**Objective:**

This observational study aims to generate hypotheses on the detection and elimination of acceleration artifacts due to the intrinsic rotation of accelerometers and their differentiation from heart-induced sensor accelerations.

**Methods:**

Multimodal data from 4 healthy participants (3 male and 1 female) using BCG-SCG and an electrocardiogram will be collected and serve as a basis for signal characterization, model modulation, and location vector derivation under parabolic flight conditions from µ*g* to 1.8*g*. The data will be obtained during a parabolic flight campaign (3 times 30 parabolas) between September 24 and July 25 (depending on the flight schedule). To detect the described acceleration artifacts, accelerometers and gyroscopes (6-degree-of-freedom sensors) will be used for measuring acceleration and angular velocities attributed to intrinsic sensor rotation. Changes in acceleration and angular velocities will be explored by conducting descriptive data analysis of resting participants sitting upright in varying gravitational states.

**Results:**

A multimodal data set will serve as a basis for research into a noninvasive and gentle method of BCG-SCG with the aid of low-noise and synchronous 3D gyroscopes and 3D acceleration sensors. Hypotheses will be generated related to detecting and eliminating acceleration artifacts due to the intrinsic rotation of accelerometers and gyroscopes (6-degree-of-freedom sensors) and their differentiation from heart-induced sensor accelerations. Data will be collected entirely and exclusively during the parabolic flights, taking place between September 2024 and July 2025. Thus, as of June 2024, no data have been collected yet. The data will be analyzed until December 2025. The results are expected to be published by June 2026.

**Conclusions:**

The study will contribute to understanding artificial acceleration bias to signal readings. It will be a first approach for a detection and elimination method.

**Trial Registration:**

Deutsches Register Klinische Studien DRKS00034402; https://drks.de/search/en/trial/DRKS00034402

**International Registered Report Identifier (IRRID):**

PRR1-10.2196/63306

## Introduction

Complex methods of cardiovascular diagnostics are increasingly being integrated from the clinical setting into everyday life. Wearable smart sensor technology necessitates real-life measurements to make the results usable for individualized and tailored care [1]. Ballistocardiography (BCG) and seismocardiography (SCG) are generally suitable for cardiovascular diagnostics (heart rhythm, functionality, and vascular status) and the extraction of parameters such as blood pressure or respiration at a relatively low cost and with a low-risk potential. They can thus provide support for diagnosing and monitoring potential heart abnormalities [2-12]. BCG and SCG are noninvasive methods for measuring oscillating recoil movements of the whole body (in the case of BCG) or the chest (in the case of SCG), caused by ballistic forces generated by the blood flow (in the case of BCG) or mechanical actions of the heart (in the case of SCG) [13]. The accelerations of these movements can be derived from digital 3D acceleration sensors (accelerometers) on the body surface [14,15]. These sensors are commonly positioned over the apex of the heart or the sternum and measured across all axes. Even though BCG-SCG has been known for several decades and despite numerous research projects, there is still a fundamental uncertainty in characterizing the SCG-BCG signals, making interpretation extremely difficult. Earth gravity (*g*=1) can influence the translational 3D acceleration measurement of SCG signals because of the rotatory movement of the sensor on the body surface. The heart beat does not only cause 3D translatoric (x, y, z) but also rotatory movements (angular velocity: x=yaw, y=pitch, and z=roll) [16]. Hence, both translatoric movements and angular velocities are in the same frequency domain of the regular SCG signal. As a result, the actual translatoric acceleration caused by the heart is affected by additional “artifacts” due to the change in orientation of the acceleration axis relative to the earth’s gravity. This can lead to BCG-SCG–like acceleration artifacts on the axes and thus to constructive or destructive interference with the actual BCG-SCG signal. These can lead to deformation and, thus, misinterpretations of the signal. Considering these artifacts, the interpretation of SCG signals is biased, so there is a strong need to obtain a clear SCG signal description so that the correlation of the SCG signal with the physiological parameters of the heart can be successful in the long term.

To characterize the BCG-SCG signals and their features as precisely as possible, clearly defined and compensated position vectors of the acceleration on the reference system “heart” are necessary. The signals can be compared with reference methods, which helps characterize BCG-SCG signals. This study aims to answer the overall question of what form the BCG-SCG signal takes when the acceleration artifacts created by the intrinsic rotation of synchronous accelerometers, measuring acceleration, and gyroscopes, measuring angular velocities (6-degree-of-freedom [6-DOF] sensors), in relation to earth’s gravity are filtered out. In this context, the question arises of what related artifacts look like and whether it is possible to eliminate them from the signal. Thus, ARTIFACTS is an observational study to generate hypotheses on detecting and eliminating such artifacts and differentiating them from heart-induced body acceleration. Against this background, ARTIFACTS aims to detect these artifacts by performing examinations in different gravitational states. This includes describing the artifacts (eg, by determining how strongly they influence the BCG-SCG signal), differentiating them from heart-induced sensor accelerations, and, following this, eliminating them from the signals. For this purpose, data on individual acceleration patterns of cardiac physiology under different gravitational influences will be collected. These data will then be descriptively analyzed concerning intra- and interindividual differences, serving as a basis for signal characterization, model modulation, and location vector derivation. One focus of the analysis will be to describe aspects related to the formation and the effects of the artifacts. Data from reference methods will be collected to help characterize the signals. Exploratively, artificial patterns will be identified and described using individually collected data and actual conditions. The data will serve as a basis for developing models for defined and compensated acceleration position vectors.

## Methods

### Overview

The study aims to collect intra- and interindividual multiaxial movement (angular velocity, measured using gyroscopes, and acceleration, measured using synchronous, using synchronous accelerometers [6-DOF sensors]) and electrocardiogram (ECG) data at different gravitational states (µ*g* to hypergravity) under physiological real-life conditions in parabolic flights. The data will be used to describe acceleration artifacts caused by the sensors’ physical rolling, yawing, and pitching movements on the body surface. ARTIFACTS is designed as an open, prospective, noninterventional, noninvasive, nonrandomized, descriptive study. The procedure, including the experiment, will be identical for all participants. For this reason, blinding is not appropriate. The study will last 10 months (September 2024 to July 2025). The data collection will take approximately 3 weeks.

### Study Objectives

The primary end point comprises the description of acceleration artifacts (morphology and expression), differences in accelerations or angular velocities, and the identification of variables that affect acceleration sensors due to gravitational acceleration. The evaluation of signal filtering and peak detection algorithms are the secondary end points of this study. Of particular interest are differences in motion trajectory resulting from SCG-BCG data to describe and quantify the influence of artifact formation, more specifically dedicated consideration of the differences in accelerations and angular velocities in 6 spatial dimensions on 2 different body locations in µ*g*, 1*g*, max 1.8*g*, and transitions.

### Hypothesis

**Figure 1:**
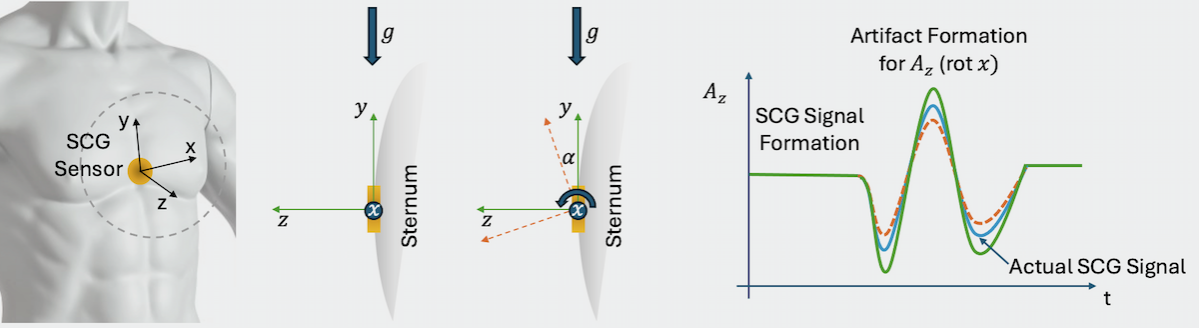
Schematic representation of the artifact formation as a basis for the derivation of the hypothesis (example for x-rotation). Due to the change in the axis position relative to the gravitational acceleration, there is a change in acceleration along this axis (here z-axis and y-axix). At g=0 the impact of gravity is ruled out and no change in acceleration due to rotation is expected (no artifacts). At hyper-g (g=1.8g) the effects of the artifacts can increase. SCG: Seismocardiography.

During the phases of hyper-*g* conditions (*g*=1.8*g*), the acceleration changes caused by rotation (artifacts) are more dominant. In the phase of 0*g* (no gravitational influence), the impact of artifacts should not be present. Figure 1 depicts an example of this artifact formation for a rotation of the sensor around x-axis.

### Participants

In ARTIFACTS, 4 healthy adult persons (male and female; 18-65 years old, fit for duty) without structural heart disease and in stable sinus rhythm will be included. Participants who are unwilling or unable to participate; who have an addiction, heart or cardiovascular disease, mental illness or panic disorders, allergy or intolerance to electrode gel or wound dressings, emphysema, and multimorbidity (ie, co-occurrence of at least 2 chronic conditions); or who are breastfeeding or pregnant will be excluded from the study. Recruitment takes place via personal contact. All study participants are members of the scientific group. Recruitment is done on a strictly voluntary basis. The project’s explorative and hypothesis-generating approach justifies the number of cases. The project is a data collection of heart-healthy individuals in the form of an observational study to generate hypotheses on detecting and eliminating acceleration artifacts due to the self-rotation of the 6-DOF sensors and their differentiation from heart-induced body acceleration. Four participants (3 male and 1 female) are sufficient for (1) evaluating the feasibility of the measuring or recording method, (2) obtaining data for postanalysis, (3) determining variation in individual biophysical vibrations related to the cardiovascular system, and (4) having sufficient backup personnel for dropouts, for example, due to sudden illness. The participants are a convenience sample of the study personnel. There are no financial expenses for the participants in this study.

### Study Procedure

The German Space Agency, Germany, initiated and organized the campaign. The study participants will be prepared for the measurements in Bordeaux, Mérignac, at the NoveSpace center. The measurements are carried out in an aircraft designed explicitly for parabolic flights as part of the parabolic flights over the Atlantic. All data will be collected in France. The total duration of a single measurement series is approximately 3.5 hours. One measurement point is planned per study participant.

**Figure 2:**
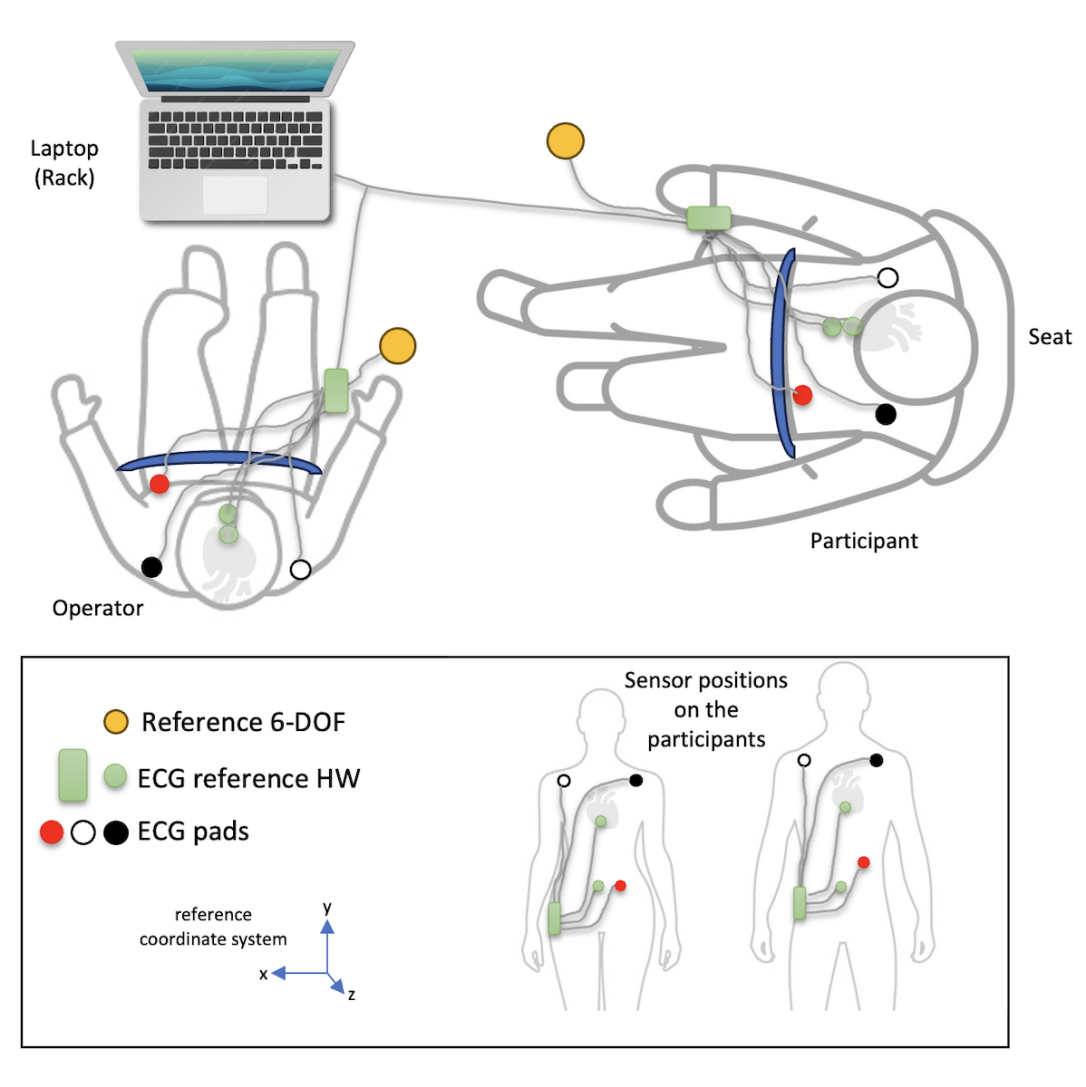
Setup, accelerometric angular velocity and ECG readings of a resting participant fixed to a seat (right) during parabolic flights. An operator manages preparation and execution of the experiments (person on the left, fixed to a sting position). Roles change during each flight. A reference accelerometer-gyroscope is fixed to the ground of the aircraft for reference. 6-DOF: 6-degree-of-freedom; ECG: electrocardiogram; HW: hardware.

The study participants will be informed in advance about the study, and their consent to participate will be obtained. A physician will obtain informed consent outside of a possible dependency relationship. After the candidates have consented to participate in the study, the information required to validate the inclusion and exclusion criteria is requested and checked. A flight physician will check the physical condition of the participants. Age, weight, height, and BMI are inquired and recorded. While still on the ground, the study participants will be outfitted with three 6-DOF sensor patches and ECG electrodes, and both types of sensors will be taped to their bodies (Figure 2). Data acquired via the patches and the ECG will be clock-synchronous. For the ECG, the Einthoven II lead will be used. For the SCG-BCG measurements, sternal and lower back positions will be used. The study participants will be transferred to the Airbus A310 Zero G and seated (Figure 1). During the experiment, participants will stay seated for the duration of the parabolic flights. The participants are asked to avoid any physical activity during the measurements. There is a one-time role change from the participant to the investigator. Participation in this study lasts a maximum of 6 days, including preparation and 1 flight day during the parabolic flight campaign. On the flight day, the participation will consist of 2 hours for preflight activities, 3.5 hours for the parabolic flight, and approximately 30 minutes for postflight follow-up after landing.

### Schedule

Three parabolic flights with 30 parabolas (+1 test parabola excluded from measurements) are planned. One parabolic flight is planned per day (see Figure 2). The participant and operator are fitted with the SCG and BCG sensor patches (sternum and lower back) and the 1-channel ECG on the ground. After entering the aircraft, the operator and test participants check the available equipment and its functionality. The positions of the reference sensors are approved. The participant is strapped to the seat during the flight and for the experiment preparation. The operator then tests connectivity and checks data recording (including the reference sensor). When all readings are satisfactory, and no adjustments are required, the operator straps himself into a sitting position. The operator starts the logging of data, and the experiment starts. After 20 parabolas, the operator and test participant switch positions and roles. After the last parabola, the logging is stopped, the data are stored and backed up, and the operator and the test participant are released from their positions. Back on the ground, the sensors and the ECG are removed, and the equipment is prepared for the next flight.

**Figure 3:**
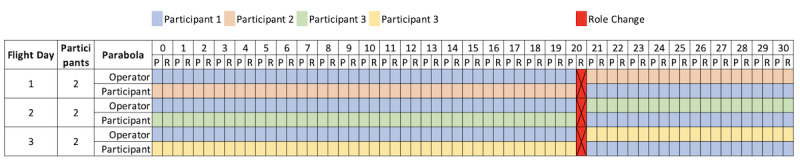
Schematic schedule for all 3 flight days and all 4 participants with 30 parabolas per flight day. A cycle comprises a parabola (P) and a rest phase (R). The role change between the operator and participant occurs after 20 parabola cycles during the resting phase.

A schematic schedule of how the 4 test participants will be used on the 3 flight days is shown in Figure 3 and Table 1. The key operator (here, participant 1) will start with the operator’s role to rectify technical problems at an early stage and obtain as much data as possible from the participant. After 20 parabolas, the role is changed, as mentioned before. The first parabola of the day is not counted and serves as the first test of the sequence. This results in the total data after 3 days of flight (the amount of data is based on the parabola cycles), as shown in Table 1.

**Table 1:** Number of measurements (parabolas) for each participant and in each role.

Role	Flight day							Sum	
	1		2		3				
	Participant	Operator	Participant	Operator	Participant	Operator	Participant		Operator
Participant 1	10	20	10	20	10	20	30		60
Participant 2	20	10	—^a^	—	—	—	20		10
Participant 3	—	—	20	10	—	—	20		10
Participant 4	—	—	—	—	20	10	20		10

^a^Not applicable.

### Measurements

Aside from sociodemographic data (sex and age), linear accelerations, and angular velocity (6-DOF SCG-BCG signals), the heart rate (ECG) and the gravity level will be recorded. The acceleration will be detected in the form of SCG-BCG signals.

### Equipment

**Figure 4:**
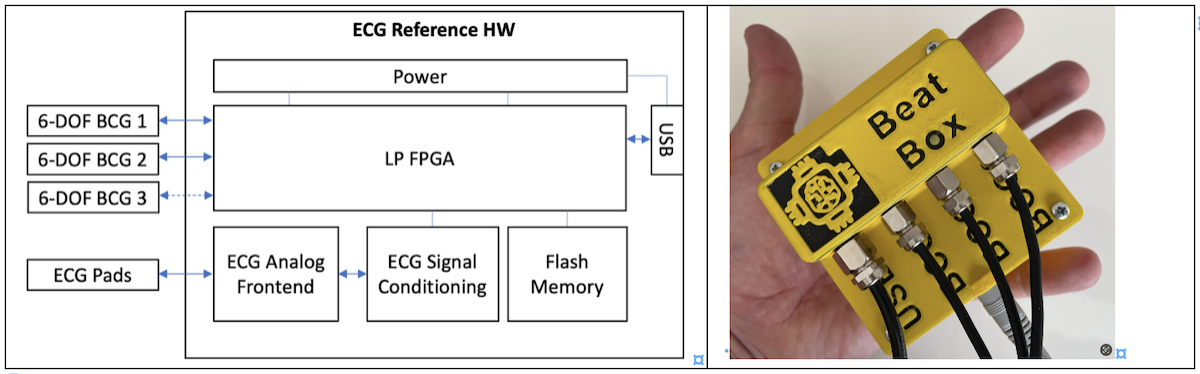
The basic structure of the measurement system is illustrated on the block diagram (left) and BCG/SCG/ECG reference hardware (right). 6-DOF: 6-degree-of-freedom; BCG: ballistocardiography; ECG: electrocardiogram; HW: hardware; LP FPGA: low power field-programmable gate array; SCG: seismocardiography.

For the measurements, a fused sensor system consisting of commercially available 3D accelerometers and 3D gyroscopes used for determining the micromovements, and an ECG, serving as a reference system, is employed. This device can simultaneously record 3 times 6-DOF SCG-BCG signals along with an ECG (all with clock synchronization), while still maintaining high precision. This system is required to investigate artifact formation precisely. To ensure high-precision and low-noise measurement, the principle of differential sensing [17] is used to make SCG-BCG data measurable with ultralow noise and also to record data for the reference coordinate system on the aircraft floor. It is crucial for the experiment that all data are recorded synchronously and with as little jitter as possible. A field-programmable gate array, whose parallel structures are very well suited for this purpose, is used for this. The basic implementation of the ECG reference hardware is based on a previous prototype [18] (see Figure 4).

### Ethical Considerations

The risk of damage to health through participation in the study is negligible and is offset by the positive benefit of the knowledge gained. There is no direct benefit for the study participants. The data collected are of great interest to the general public, for example, for further scientific studies of health monitoring systems and possible technology transfer. There is no therapeutic intervention. The principal risk associated with parabolic flights is the risk of an aeronautical accident. This risk has been assessed by the technical experts of the European Aviation Safety Agency, who concluded that these flights provide an acceptable level of safety. The study-related examinations are all painless and noninvasive. There is minimal risk in conducting the study due to very rare allergic reactions to the electrode gel or wound dressing. The risk of damage to the participants’ health through participation in the study is negligible (allergic reactions to electrode gel, symptomatic motion sickness, and pressure effects in the middle ear are all rare occurrences). The positive benefit of the knowledge gained offsets these risks. Participation in the study has no consequences for further clinical treatment. The main undesirable effect may be travel sickness (nausea and vomiting) due to the succession of variations in gravity levels. The clinical course of the adverse event will be followed according to accepted standards of medical practice, even after the end of the observation period, until a satisfactory explanation for the adverse event is found or the investigator considers it medically justifiable to terminate follow-up. In case of motion sickness, the experiment is stopped, and the participant returns to a seat under the supervision of the onboard doctor. Once the aircraft has taken off, it is impossible to get off the plane, and the participant will thus have to endure the remaining flight. Neither interactions with concomitant medical treatments nor serious adverse events, including serious health hazards and severe effects of the measurement devices or reportable adverse events, are expected.

Freedom of publication is guaranteed. All project members will be informed before the study findings are used and published. All publications based on the data obtained will be coauthored by both parties in accordance with the DFG (German Research Foundation) criteria.

The study’s objectivity can be guaranteed through critical consideration and the comprehensive and targeted division of the implementation, analysis, support, and monitoring by unbiased study staff, as well as compliance with the premise of the 2-eye principle and universal study transparency.

As stated, the participants are part of the study personnel. The study participants do not receive any separate remuneration.

ARTIFACTS was approved by the Ethics Committee of the Ärztekammer Westfalen-Lippe and WWU Münster, Germany, on May 13, 2024 (2024-081-f-S; Chairman: Prof Dr WE Berdel).

### Informed Consent

Participants’ informed consent is mandatory for study participation. Volunteers will receive an electronic version of the experiment information file several weeks before the parabolic flight campaign. This document will detail the experiment’s purpose, methods, and duration. By the day before the flight, volunteers will be given a paper version of the experiment information file, meet the research team, tour the aircraft, and become acquainted with the procedures and devices used in the experiment. Potential risks and constraints will be explained, and volunteers will have the opportunity to ask questions. They will have at least 2 hours to review the information before signing the informed consent form. After this, an enrollment visit will occur, and volunteers will be further familiarized with the procedures.

### Safety

Only tried and tested measurement system components are used to prevent any risk of impairment or injury to the study participants. Furthermore, all measurements concerning the study participants will be done by trained personnel. The technology, which is used directly on the study participants’ bodies, works exclusively with low voltage that is harmless to health. The devices are fully insulated. The measurement can be terminated at any time by the study personnel or participants. The health risk of participating in studies is negligible. In the event of study-related damage, all participants in a flight are covered by insurance.

### Data Collection

All data, including electronically stored data and hard copies, will be pseudonymized for the purposes of the research. Data collection and documentation are primarily carried out electronically on an encrypted and password-protected research laptop. The data records are then transferred to the secure server infrastructure (research area) at Bielefeld University and Hamburg University of Technology (TUHH). An authorization and role concept regulates access to the research data. The password-protected pseudonymization list is stored separately on an encrypted data carrier and physically stored in a suitable, lockable cabinet. Only the study management has access to this list and the data. The data are analyzed anonymously. Information related to data security and protection can be found in the corresponding section in the provided information material. Data protection is taken into account in accordance with the applicable laws. The European Union General Data Protection Regulation (EU-GDPR) is relevant for the European area. The legal basis for the collection, storage, processing, and transfer is the informed written consent of the study participants. Retention and deletion periods are based on the GDPR; the same applies to the data participants’ rights to information. The data collected during this study will be stored for at least 10 years after the completion or discontinuation of the study following DFG regulations. Research data management is implemented to handle research data, taking into account the guidelines for handling research data at TUHH and the research data policy of Bielefeld University. The existing infrastructure of both universities is used to ensure long-term storage within the permitted time limits. All data used as a basis for publications will be made available anonymously together with the publication.

### Analysis

Primarily, no inferential statistics are planned. In the early research stage, the focus is on descriptive questions. The description is carried out by determining minima and maxima, mean value, SD and variance, median and IQR, correlations, interrater stability, and signal-to-noise ratio. In the event of a termination of data collection due to external circumstances such as air sickness, all data recorded up to that point are analyzed. Multiple imputation compensates for missing values.

Statistical analyses will be carried out using R software (version 4.4.1 or later, open source; R Project for Statistical Computing) and Python (version 3.12.4 or later, open source; Python Software Foundation). The research team will carry out the data analysis.

## Results

ARTIFACTS is an experiment within the AuRelia project. AuRelia has been funded since August 2023 by the German Federal Ministry of Economics and Climate, funding code FKZ:50RP2350. Additional institutional resources are used.

The data will be collected entirely and exclusively during the parabolic flights, which will take place between September 2024 and July 2025. As of June 2024, no data sets have been collected yet. Data analysis will take place until December 2025. The publication of results is planned for June 2026.

A total of 4 participants have been recruited (3 male and 1 female). The study’s primary end point involves characterizing acceleration artifacts, including their morphology and expression, analyzing variations in acceleration, and identifying factors influencing acceleration sensors under gravitational acceleration. The secondary end points encompass the assessment of signal filtering and peak detection algorithms. A multimodal data set will be available that serves as the foundation for research into a noninvasive and gentle method of BCG-SCG using 6-DOF sensors (3D gyroscopes and 3D accelerometers). This research explores hypotheses regarding the formation, detection, and mitigation of acceleration artifacts arising from the intrinsic rotation of BCG-SCG sensors and their differentiation from heart-induced sensor accelerations. The collection of the multimodal basic data set provides the necessary basis for research into a noninvasive and gentle method of BCG-SCG with the aid of low-noise and synchronous 6-DOF sensors. This research contributes fundamental findings for unambiguous characterization based on compensated location vectors. It also helps develop methods for integrating signal characterization on resource-limited sensors and translational models for mapping between BCG-SCG and reference methods.

## Discussion

### Prior Work

To our knowledge, prior work on BCG-SCG signals has not considered acceleration bias caused by gravitational influences on the self-rotation of acceleration sensors. However, we found several studies in our literature that address the participant of motion artifacts in BCG and potential countermeasures [19-21]. Also, we identified 2 publications considering BCG measurements under parabolic flight conditions, which address highly artifact-biased data sets but without explaining the kind or source for the artifacts [22,23].

### Anticipated Findings

This study will have shown that artifacts in BCG-SCG signals caused by the roll, pitch, and yaw movements of accelerometers in relation to the earth’s gravity can be detected and eliminated from BCG-SCG signals, and these signals can thus be characterized more precisely. In the context of the use of multimodal measurement systems in this study, uncertainties in using these are expected to become apparent. However, this demonstration is not the aim of the study. Thus, the study provides insights into the uncertainty of multimodal measurement systems.

### Interpretations and Implications

Our study will provide a basis for using more clearly characterized BCG-SCG signals in future studies and applications. In future studies in which BCG-SCG signals are to be evaluated, more reliable evaluations can thus be made. The precise signal characterization will also be of clinical relevance in the future. According to [24], monitoring by SCG and associated applications is still only possible to a very limited extent due to artifacts in the signal. Through a good characterization of BCG-SCG signals, BCG-SCG could be used more reliably than before for the diagnosis of diseases such as heart disease and the monitoring of heart health. Based on the findings of this study, clinical applications for diagnostics using BCG-SCG can be further developed.

### Strengths and Limitations

A strength of this study is that investigating artifacts in BCG-SCG signals using different gravity states is a pragmatic approach to improving signal characterization. A small sample is sufficient to obtain fundamental findings. Matching BCG-SCG signals with reference methods such as ECG is fundamental for subsequent studies on how ECG and BCG-SCG can complement each other. Although it is assumed that the scope of the study is sufficient to characterize the BCG-SCG signals as planned, even longer sensory measurements in different gravitational states could possibly further improve signal characterization. In this study, the accelerations caused by the movement of the accelerometers in relation to the earth’s gravity are distinguished. Generally, valid statements about heart-induced sensor accelerations cannot be made because of the small and diverse sample size. However, because this is not the aim of this study, it is not a limitation in the strict sense.

### Future Directions

Future studies can test how well the findings from this study regarding the detection and removal of artifacts can be transferred to BCG-SCG signals recorded over more extended periods under normal terrestrial gravity conditions. In addition, various test conditions can be used to investigate how precisely the artifacts can be detected and how they can be removed from the signal, even when the respective sensor is in different positions due to various everyday body postures (such as lying down or bending over). In addition to the artifacts examined in this study, artifacts may arise from other sources (eg, internal body movements caused by speech or external movements such as passing vehicles). Artifacts coming from different sources are not always easy to distinguish. As a result, it is not always possible to identify which source leads to strong artifacts. Knowing this may help determine under which circumstances BCG-SCG signals can be best characterized and thus should be recorded, which could be investigated in future studies.

### Conclusions

ARTIFACTS is fundamental research, the findings of which will contribute to precise signal characterization based on compensated position vectors. It also provides insights into the uncertainties of multimodal measurement systems and develops methods for integrating characterization on resource-limited sensors and translational models for mapping between BCG-SCG and reference methods, namely, echocardiography, phonocardiography, and electrocardiography. Taken together, a portfolio of methods will be available as a scientific basis for further research to enable cost-effective wearables with a wide range of applications for outpatient diagnostics and monitoring in prevention, health promotion, and diagnostics.

## Abbreviations

BCG: ballistocardiography
DFG: German Research Foundation
6-DOF: 6 degrees of freedom
ECG: electrocardiogram
GDPR: General Data Protection Regulation
SCG: seismocardiography
TUHH: Hamburg University of Technology

## Data Availability

The data sets generated during and/or analyzed during this study are available from the corresponding author on reasonable request. The full data sets are, however, not publicly available due to restrictions as they contain information that could compromise the privacy of research participants.
